# Measuring What Matters: RECIST Response Does Not Predict HRQoL in Early-Phase Clinical Trials

**DOI:** 10.3390/cancers18081242

**Published:** 2026-04-14

**Authors:** Jessie Nguyen, Udit Nindra, Joanne Tang, Walid Zwieky, Jun Hee Hong, Martin Hong, Joseph Descallar, Andrew Killen, Adam Cooper, Kate Wilkinson, Abhijit Pal, Christina Teng, Aflah Roohullah, Joe Wei, Weng Ng, Charlotte Lemech, Wei Chua

**Affiliations:** 1 Department of Medical Oncology, Liverpool Hospital, Liverpool, NSW 2170, Australia; joanne.tang@sa.gov.au (J.T.); walid.zwieky@health.nsw.gov.au (W.Z.); junhee.hong@health.nsw.gov.au (J.H.H.); martin.hong@health.nsw.gov.au (M.H.); adam.cooper@health.nsw.gov.au (A.C.); kate.wilkinson1@health.nsw.gov.au (K.W.); abhijit.pal@health.nsw.gov.au (A.P.); aflah.roohullah@health.nsw.gov.au (A.R.); weng.ng@health.nsw.gov.au (W.N.); wei.chua@health.nsw.gov.au (W.C.); 2 Ingham Institute for Applied Medical Research, Liverpool, NSW 2170, Australia; joseph.descallar@health.nsw.gov.au; 3 Scientia Clinical Research, Randwick, NSW 2031, Australiachristina.teng@scientiaclinicalresearch.com.au (C.T.); charlotte.lemech@scientiaclinicalresearch.com.au (C.L.); 4 School of Medicine, Western Sydney University, Penrith, NSW 2751, Australia

**Keywords:** early phase clinical trial, HRQoL, RECIST, patient-reported outcomes

## Abstract

**Background/Objectives**: Early-phase clinical trials (EPCTs) focus on safety and preliminary efficacy, often assessed by RECIST (Response Evaluation Criteria in Solid Tumours) tumour response. Health-related quality of life (HRQoL) is rarely evaluated in EPCTs and may not align with radiological outcomes. **Methods**: The PEARLER (Patient Experience in Early-Phase Cancer Clinical Trials) study evaluated the demographics, tumour response, HRQoL, and therapy type in two early-phase trial units in Australia between August 2023 and 2024. Patients completed the EORTC QLQ-C30 at baseline and follow-ups. The Global Health Status (GHS) score was selected as the primary HRQoL measure. Tumour response was assessed using RECIST 1.1. Spearman correlation and Kruskal–Wallis testing assessed the associations between RECIST, cross-sectional GHS change (ΔGHS; follow-up minus baseline), and therapy types. Multilevel models were used to evaluate longitudinal GHS values per RECIST category. **Results**: Of 122 patients recruited to the PEARLER study, 74 patients had paired RECIST and HRQoL data (complete response (CR) *n* = 0; partial response (PR) *n* = 15; stable disease (SD) *n* = 39; progressive disease (PD) *n* = 20). The median change in GHS was zero across RECIST groups, with broad individual variability. Notably, 18 of 54 patients (33.3%) with stable or responding disease experienced HRQoL decline. Meanwhile, 10 of 20 (50%) patients with PD experienced stable or improving HRQoL. The best RECIST response and ΔGHS showed a weak but statistically significant negative relationship (Spearman ρ = −0.28, *p* = 0.017), with the Kruskal–Wallis test demonstrating χ^2^ = 6.20 (*p* = 0.045), indicating modest group differences driven by the deterioration in PD patients. The multilevel model demonstrated a lower GHS in patients with PD, with no statistically significant interaction of GHS change over time with the RECIST response (*p* = 0.226). **Conclusions**: HRQoL change is largely independent of radiologic tumour response and therapy type in EPCT participants. Patients experienced a HRQoL decline despite tumour response. Incorporating patient-reported outcomes alongside RECIST and safety outcomes is important to fully capture the impact of investigational therapies and guide patient-centred trial designs.

## Simple Summary

In early-phase cancer research, which assesses new cancer therapy, the detection of tumour shrinkage and drug safety are key findings of interest, evaluated through escalating drug dosages. Given the early drug testing phase, not all participants would derive benefit, with a limited number having cancer control, whilst many may sustain side effects with a risk of compromising their quality of life and function. It is uncertain whether changes in tumour size, as a measure of cancer control, would be associated with meaningful changes in the quality of life in participants of early-phase cancer research. This study found discordance between tumour control on imaging and patients’ lived experiences of treatment. One in three participants with a stable or reduced tumour size reported worsening wellbeing; meanwhile, half of the patients had improved wellbeing scores despite tumour growth. This result may assist researchers in adapting trial designs to better align with the needs and values of cancer patients and to improve the quality of early-phase cancer research.

## 1. Introduction

Oncology early-phase clinical trials (EPCTs) have evolved from toxicity-focused investigations into multidimensional studies exploring safety, pharmacokinetics, pharmacodynamics, and preliminary efficacy signals. Response Evaluation Criteria in Solid Tumours (RECIST) provides a reproducible framework for quantifying tumour shrinkage as an efficacy assessment for EPCT investigational products [1]. Tumour regression on imaging does not always translate into improved symptoms, physical function, or overall wellbeing, nor is it necessarily representative of survival outcomes [2]. Radiological changes capture only one aspect of a patient’s cancer goal. Similarly, the Common Terminology Criteria for Adverse Events (CTCAE) is commonly used for adverse events (AEs) reporting by clinicians, which may not reflect the patient’s experience and severity of symptoms [3].

The use of patient-reported outcomes (PRO) and health-related quality of life (HRQoL) as key endpoints in clinical trials is becoming more common in clinical trials, as it provides a complementary perspective to clinical benefit, representing patients’ subjective evaluations of their function [4]. HRQoL is often assessed through validated patient-reported outcome tools such as the EORTC QLQ-C30 and the FACT-G [5,6]. PRO-CTCAE is a complementary tool to CTCAE, self-reported by patients to capture symptomatic AEs in line with CTCAE items [7]. Incorporating PROs into oncology trials is an important consideration for drug regulatory approvals and is endorsed by professional oncological societies [8,9,10]. However, HRQoL remains under-reported in EPCTs [11,12]. Fewer than 2% of published phase I studies report longitudinal PRO data, and when collected, the results are often not analysed or reported systematically [11].

Evidence from later-phase trials demonstrates that HRQoL is not directly correlated to survival outcomes, and its trajectories often diverge from subjective tumour response [6,13]. This discordance likely reflects the complex interplay of drug-related toxicity, symptoms of advanced cancer, and the psychosocial effects of cancer burden and therapies [1,14].

It is unclear whether improvement in tumour burden corresponds to HRQoL in EPCTs’ participants. An understanding of whether radiological responses translate into meaningful HRQoL improvement is essential for clinical decision-making, with practical implications for trial design. If radiologic and patient-reported outcomes are poorly correlated, then both should be captured to ensure that therapeutic value is measured not only by imaging, but also by the outcomes important to patients, such as patient-reported outcomes.

The PEARLER (Patient Experience in eARLy phasE tRials) study was established as a prospective, multicentre cohort study capturing HRQoL and non-clinical outcomes, including financial, social, and time burdens in patients undergoing EPCTs. Earlier analyses from PEARLER showed that, overall, HRQoL remained stable across trial cycles and that financial, social, and time toxicities were prevalent but generally remained stable during participation [15,16].

This sub-study analysis of the PEARLER study specifically assesses the relationship between radiologic response and HRQoL change for patients undergoing EPCTs. We hypothesised that the RECIST response status would show limited alignment with patient-reported global health scores, reflecting the divergence between objective and subjective treatment outcomes in this context.

## 2. Materials and Methods

### 2.1. Study Design and Participants

This prospective observational sub-study was nested within the PEARLER (Patient Experience in eARLy phasE tRials) study, which evaluates the patient’s experience of participation in early-phase oncology clinical trials. This study was conducted across two major Australian EPCT units: the Department of Medical Oncology, Liverpool Hospital, and Scientia Clinical Research, Randwick. Participants were adults ≥18 years with histologically confirmed malignancy, who had consented to enrol in phase I oncology trials between August 2023 and August 2024. Trials included both first-in-human and dose-expansion studies of targeted therapies and immuno-oncology agents.

Eligibility for this sub-analysis required the availability of paired EORTC QLQ-C30 questionnaires (baseline and at least one follow-up) and radiologic assessment according to the Response Evaluation Criteria in Solid Tumours (RECIST 1.1). Participants without contemporaneous imaging or incomplete questionnaires were excluded. No restrictions were placed on the tumour type, prior therapy, or investigational treatment.

Sociodemographic and clinical variables, including age, sex, tumour type, and treatment class, were obtained from trial records and study databases rather than separate participant-completed questionnaires.

HRQoL assessments were performed at baseline (prior to or at commencement of trial therapy) and at each trial treatment cycle’s Day 1. For this sub-study, up to a maximum of six cycles were included, given that a prior institutional cohort’s results demonstrated that the majority of EPCT patients completed less than six cycles [15].

HRQoL questionnaires using the EORTC QLQ-C30 questionnaires were provided in printed form (Supplementary File S1) at routine clinic visits within the early-phase trial units [6]. Data collection was coordinated by trained research staff at each participating site. Participants completed questionnaires either independently or with minimal assistance if required. Completion time was approximately 5–10 min. Questionnaires were administered in a standardised manner across both sites, with efforts made to minimise missing data by aligning completion with scheduled trial visits. Where assistance was required (e.g., due to visual or physical limitations), this was limited to clarification of questions without influencing responses.

### 2.2. Radiologic Response and HRQoL Assessment

Tumour response was evaluated by site investigators using the RECIST 1.1 criteria and categorised as a complete response (CR), partial response (PR), stable disease (SD), or progressive disease (PD) [1]. Imaging assessments were scheduled per parent-trial protocols, typically every 6–8 weeks.

Health-related quality of life (HRQoL) was measured using the European Organisation for Research and Treatment of Cancer Quality of Life Questionnaire Core 30 (EORTC QLQ-C30) version 3.0 [6]. This validated 30-item tool captures multiple functional (physical, role, cognitive, emotional, and social) and symptom domains, along with a two-item Global Health Status (GHS) scale. The full analysis of HRQoL of the entire PEARLER cohort was reported in a prior publication [15]. This study selected GHS as the primary HRQoL measure.

The GHS score is assessed by 2 items of the EORTC QLQ-C30: Item 29 “How would you rate your overall health during the past week?”; and Item 30 “How would you rate your overall quality of life during the past week?”. Responses were transformed to scores following the EORTC scoring manual, with higher scores indicating a better global quality of life. GHS change (ΔGHS) from baseline was calculated at predefined follow-up assessments on Day 1 of each treatment cycle (up to six cycles). A change of ≥10 points was prespecified as clinically meaningful, consistent with established interpretive thresholds [4]. GHS scores across multiple cycles (Day 1 of each cycle, up to 6 cycles) were also reported and utilised in exploratory longitudinal analyses. For each participant, HRQoL and radiologic assessments were temporally aligned using a ±14-day window to ensure that both measures reflected the same disease interval.

### 2.3. Statistical Analysis

Given the observational design of this sub-study, the potential for confounding by clinical variables (including age, sex, tumour type, prior therapies, and trial therapy class) was recognised. However, the modest sample size and exploratory nature of the analysis limited the feasibility of fully adjusted multivariable modelling without risk of overfitting.

Prior to analysis, variable distributions were assessed using summary statistics and visual inspection. The change in Global Health Status (ΔGHS) between baseline and the best RECIST timepoint demonstrated a non-normal distribution with substantial variability. In addition, RECIST response represents an ordinal categorical variable. Accordingly, non-parametric statistical methods were selected a priori as the primary analytical approach, given their robustness to distributional assumptions and suitability for small sample sizes.

Spearman’s rank correlation coefficient (ρ) was used to assess the monotonic association between RECIST response and ΔGHS. Differences in ΔGHS across RECIST response categories (CR, PR, SD, PD) were evaluated using the Kruskal–Wallis test. For comparisons between two groups (e.g., therapy class), the Wilcoxon rank-sum test was applied. A two-sided *p*-value < 0.05 was considered statistically significant.

Multilevel linear models were additionally constructed as exploratory analyses to evaluate longitudinal trajectories of GHS over time and to account for repeated measures within individuals. Variables included the best RECIST response, cycle, and a RECIST-by-cycle interaction term. Age, sex, and therapy class were included as covariates. Given the limited sample size, these adjusted analyses were interpreted as hypothesis-generating.

Sensitivity analyses excluded patients with early withdrawal (<2 cycles) to assess the robustness of observed associations. Missing data were assumed to be missing at random and were not imputed, given the low proportion of incomplete questionnaires (<5%). All analyses were conducted in accordance with the Strengthening the Reporting of Observational Studies in Epidemiology (STROBE) guidelines.

### 2.4. Ethics Approval

This study was approved by the Sydney South West Local Health District Human Research Ethics Committee (2023/ETH00786), and all participants provided written informed consent. Research was conducted consistent with the Declaration of Helsinki and Good Clinical Practice (GCP) guidelines.

## 3. Results

### 3.1. Cohort Characteristics

Between August 2023 and August 2024, 107 out of 122 participants in the PEARLER cohort completed EORTC QLQ-C30 questionnaires. Of these, 74 had corresponding radiologic evaluations suitable for RECIST-based analysis. The median age for this cohort was 64 years (IQR 53.5–69.8; range 25–83), and 31 participants (42%) were female (Table 1). The most common underlying primary tumour types were as follows: lung (24%), gynaecological (19%), colorectal (12%), upper gastrointestinal (12%), head and neck (11%), and CNS (7%). The median number of cycles of this sample was 5 (IQR 3–6; range 2–6).

The therapeutic landscape reflected the current early-phase clinical trials environment: 45 patients (60%) received targeted agents (e.g., small-molecule inhibitors or antibody-drug conjugates) and 29 (39%) received immuno-oncology agents. The distribution of best radiologic response by RECIST 1.1 was as follows: complete response (CR) *n* = 0; partial response (PR) *n* = 15 (20.3%); stable disease (SD) *n* = 39 (52.7%); progressive disease (PD) *n* = 20 (27%) (Table 1). The baseline median Global Health Status (GHS) was 75 (interquartile range 66.7–83.3), consistent with the relatively preserved wellbeing of this cohort.

### 3.2. Overall Change in HRQoL

The median GHS change (ΔGHS) was 0, and the mean GHS change was −4.2 in this cohort (IQR −16.7 to +8.3). A total of 27% participants (*n* = 20) experienced clinically meaningful deterioration (ΔGHS ≤ −10) and 21.6% (*n* = 16) experienced clinically meaningful improvement (ΔGHS ≥ +10).

### 3.3. Relationship Between RECIST Response and GHS

**Figure 1:**
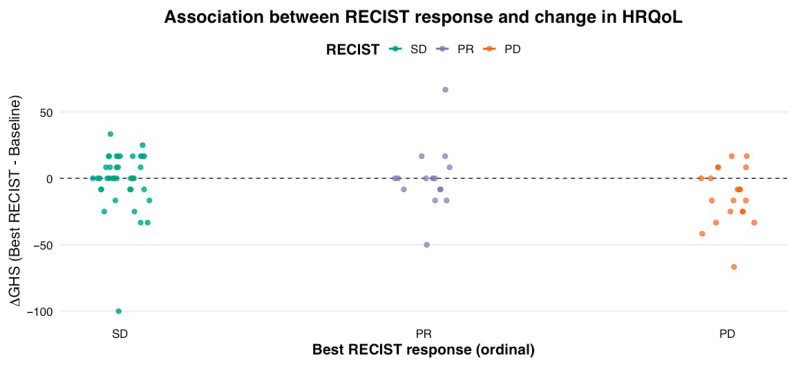
RECIST response and change in Global Health Status (ΔGHS).

Global Health Status trajectories were highly heterogeneous across RECIST categories. Median ΔGHS (each cycle follow-up—baseline) was 0.0 for SD, 0.00 for PR, and −12.5 for PD (Figure 1). Individual patient changes ranged widely from −100 to +66.7 points, indicating considerable inter-patient variability independent of radiologic outcome.

A weak inverse correlation was observed between RECIST category and ΔGHS (Spearman ρ = −0.28, *p* = 0.0017), suggesting that a greater radiologic response did not correspond to improved HRQoL and, in some instances, was associated with a modest decline. When comparing groups non-parametrically, the Kruskal–Wallis test demonstrated a modest group difference among PR, SD, and PD categories (χ^2^ = 6.20, *p* = 0.045), driven mainly by GHS deterioration in PD patients. The directionality of median HRQoL change was similar across therapy classes. Participants treated with targeted therapies had a median ΔGHS of 0.0 (IQR −16.7 to 8.3), compared with 0.0 (IQR −8.3 to 16.7) among those receiving immuno-oncology agents, with no statistically significant difference observed (Wilcoxon *p* = 0.20), indicating that the mechanism of action did not meaningfully influence quality of life changes in this cohort. The detailed distributional characteristics are provided in Table S1 (Supplementary Materials).

**Figure 2:**
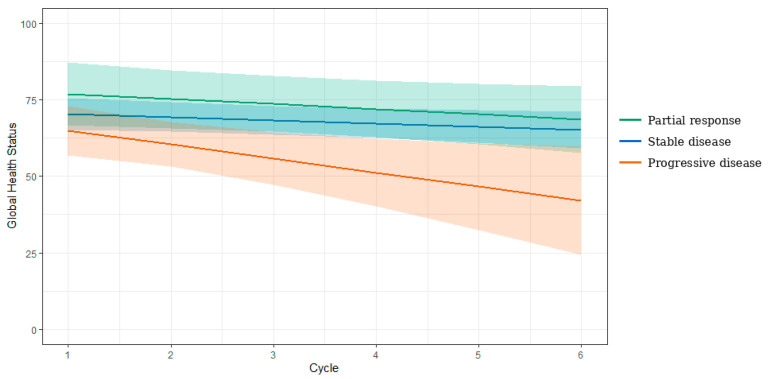
Trajectory of GHS over time by best RECIST category based on multilevel model.

The final multilevel model demonstrated a non-significant interaction between cycle and best RECIST category (*p* = 0.226). A small decline in GHS over time was observed within each RECIST group (Figure 2). In patients who received more than one cycle, from cycle two onwards, statistically significant reductions in GHS were observed in patients with PD compared with those with SD and PR (Figure 2). Model-derived estimates, including adjusted means and 95% confidence intervals, are presented in Table S2 (Supplementary Materials).

### 3.4. Longitudinal HRQoL Patterns

**Figure 3:**
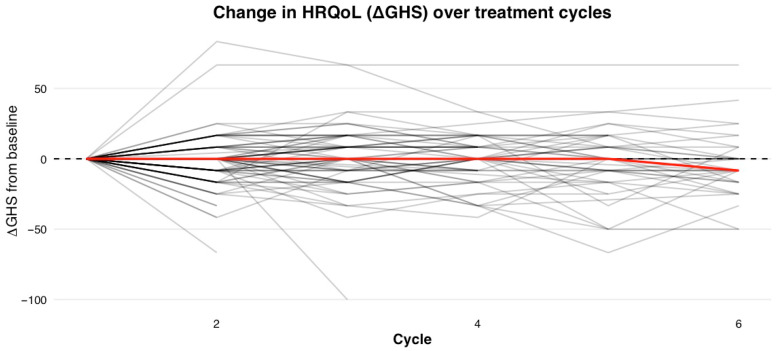
Longitudinal value of health-related quality of life. Grey—value of ΔGHS, red—median.

Longitudinal HRQoL trajectories across patients in the cohort demonstrated substantial variability over time. Although the median cohort trajectory remained stable, individual patients exhibited dynamic fluctuations in GHS (Figure 3).

### 3.5. Discordance Between HRQoL and RECIST Outcomes

**Figure 4:**
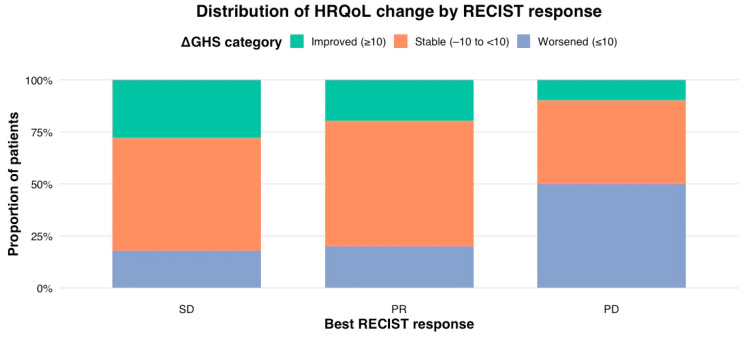
Clinically meaningful change of GHS by different RECIST response categories.

When examined categorically, discrepancies between radiologic and patient-reported outcomes were evident. Among the 54 patients who achieved SD or PR, 18 patients (33.3%) still experienced a clinically meaningful decline in GHS (≥10-point decrease). Patients with PD experienced a worse HRQoL compared to the SD or PR groups (Figure 4). Despite this, 50% (*n* = 10) reported either a stable or improved HRQoL. This discordance highlights that a subset of participants maintained subjective wellbeing despite objective progression, whereas others experienced a worsening HRQoL despite measurable tumour shrinkage.

Exploratory subgroup analysis indicated that younger participants (<60 years) may experience a greater HRQoL deterioration than older patients (median ΔGHS −16.7 vs. 0.0; Wilcoxon rank-sum *p* = 0.013), though this finding should be interpreted cautiously given the exploratory nature of the analysis.

### 3.6. Sensitivity Analyses and Data Integrity

Sensitivity analyses were undertaken to assess the robustness of the primary findings. Exclusion of patients who discontinued the parent trial prior to completing two treatment cycles (*n* = 8) did not materially alter the direction or magnitude of the observed associations between the RECIST response and HRQoL change, with correlation coefficients remaining weak and of similar magnitude. Re-analysis using alternative RECIST coding strategies, including numeric rather than ordinal categorisation, likewise yielded comparably low correlation estimates, reinforcing the consistency of the findings.

Rates of missing data were low, with fewer than 5% of observations missing for any core variable. Missing questionnaire data occurred primarily due to administrative scheduling conflicts rather than participant refusal or deterioration, supporting the assumption that data were missing at random. Visual inspection of individual HRQoL trajectories demonstrated substantial within-patient variability over time, without evidence of systematic bias related to treatment class or response category.

Overall, these analyses indicate that radiologic response, as defined by conventional RECIST criteria, is not directly reflective of a contemporaneous HRQoL change in participants enrolled in early-phase oncology trials. Despite the procedural intensity and treatment-related burden associated with EPCTs participation, the majority of patients exhibited stable or minimally changed GHS scores across treatment cycles, and objective tumour control did not predict subjective benefits.

## 4. Discussion

This analysis of the PEARLER cohort demonstrates that radiologic tumour response, as measured by RECIST 1.1, is not a reliable predictor of patient-reported health-related quality of life (HRQoL) in participants in early-phase oncology trials. Despite objective tumour stability or shrinkage, approximately one in three participants reported a clinically meaningful decline in global health status. Conversely, a notable subset of patients with progressive disease described experiencing stable or even improved HRQoL. These findings highlight the discordance between objective measures of disease control and patients’ lived experience of therapy, reinforcing the importance of integrating PROs into early-phase trial evaluation.

Phase I trials traditionally centre on dose finding and exploring early signals of anti-tumour activity, via dose-escalation and dose-expansion designs, with RECIST remaining a cornerstone for standardised response assessment. While RECIST enables reproducible comparisons, its focus on tumour size and morphology limits its capacity to capture the multidimensional impacts of treatment. Our findings are consistent with prior research showing that tumour response and HRQoL often diverge in advanced cancer [4]. The lack of proportional correlation between RECIST response and change in global health status suggests that gains associated with tumour regression may be offset by other factors. These include treatment-related toxicity, symptom burden of advanced cancer, or complex psycho-socio-emotional aspects [13,17]. This aligns with reports from later-phase immunotherapy and targeted therapy studies, where patients with partial responses frequently experience persistent fatigue, cognitive decline, or emotional strain despite disease control [18,19]. On the other hand, biologic or immune activity without measurable shrinkage can provide symptomatic improvement through decreased tumour metabolism or inflammatory modulation [20].

Beyond treatment-related factors, the demands of EPCT participation, including frequent hospital visits, prolonged observation periods, and procedural intensity, constituted a “time toxicity” burden [21]. Our earlier PEARLER analyses confirmed that financial, social, and time burdens are substantial across EPCT cycles despite remaining stable over time [16]. Psychological framing also influences HRQoL. Hope, altruism, and perceived access to new therapies may temporarily buffer distress, while disease progression may trigger resilience or acceptance mechanisms that stabilise HRQoL scores [14,22]. This phenomenon of “response shift”, where patients recalibrate their internal standards and expectations, is observed in longitudinal PRO research and likely contributes to the dissociation between radiologic and subjective endpoints [13,23,24].

These findings have important implications for EPCT design. Several groups have advocated for a multidimensional efficacy framework in assessing clinical trials’ benefit via combining objective tumour control (RECIST) with subjective wellbeing (PROs) [10,25,26]. Such approaches could be complementary in clinical decisions of dose escalation and dose selection for the expansion phases of EPCTs [10,26]. A combined endpoint could also enhance the ethical justification of continual clinical trial enrolment, particularly if marginal tumour shrinkage occurs alongside significant deterioration in HRQoL. Furthermore, whilst trials that assess PFS benefit or tumour response alone, such as EPCT, may not necessarily be associated with improvement in QoL, later-phase trials found that OS benefit and HRQoL may be correlated. Notably, better baseline HRQoL and sustained HRQoLs over the cycles have been shown to correspond with improved survival across multiple tumour types, suggesting that patient-centred endpoints may have prognostic and ethical significance [27,28].

In a broader context, our results are concordant with previous research. Durbin et al. reported that financial and emotional stress remained stable across EPCT participation in a US cohort and were not correlated with tumour response [29]. Similarly, Basch et al. reported that routine electronic PRO monitoring improved symptom control and was associated with extended survival, independent of imaging results [24,30]. Together with our PEARLER study, the above-mentioned studies reinforce the concept that patients’ experience during early-phase research is multifaceted and cannot be fully explained by tumour dynamics alone [15].

International initiatives, such as the Standard Protocol Items: Recommendations for Interventional Trials (SPIRIT)-PRO extension and the International Society for Quality of Life Research (ISOQOL) reporting guidelines, now recommend systematic collection and transparent reporting of PRO data across all clinical trial phases [31,32]. Incorporating these frameworks into the phase I design is feasible, particularly with digital data capture tools and minimal-burden questionnaires. The feasibility and acceptability of ePROs in early-phase units are increasingly supported by pilot studies demonstrating high compliance rates (>85%) and minimal impacts on workflow [33].

The strengths of this sub-study include its prospective design, systematic use of validated HRQoL measures, and inclusion of targeted and immuno-oncology therapies, reflecting the modern EPCT landscape. To our knowledge, this is the first study assessing the relationship of RECIST and HRQoL in EPCTs. The temporal alignment of HRQoL and RECIST assessments strengthens the internal validity of observed correlations. Importantly, data collection spanned two independent Australian centres, enhancing generalisability across different institutional settings. However, our study has several limitations warranting discussion. The sample size, while adequate for correlation analysis, limits statistical power for subgroup comparisons. The parent trials were heterogeneous with respect to drug mechanisms and schedules, which may introduce unmeasured confounding. Variability in HRQoL and radiologic assessment timing across protocols may reduce temporal precision. Finally, although the EORTC QLQ-C30 and, in particular, using the GHS as the primary endpoint for HRQoL assessment provide a broad measure of patients’ wellbeing, disease-specific instruments may have greater sensitivity to subtle functional changes in particular cancer types. Despite these caveats, sensitivity analyses confirmed the robustness of our primary findings.

The findings from this study support several potential applications for early-phase trial design and conduct. Future early-phase protocols should prospectively embed PRO assessments as core, rather than optional endpoints, to capture patients’ experiences in EPCTs. Moreover, combined RECIST + PRO endpoints could be assessed as exploratory efficacy measures. A careful selection of a PRO tool most relevant to clinical trials should be considered, for instance, EORTC QLQ-C30, FACT-G, PROMIS, or PRO-CTCAE [5,7,34]. These endpoints could be of major interest for regulatory consideration, aligning with the emphasis on patient-centred drug development by the Food and Drug Administration (FDA) and the European Medicines Agency (EMA) [35,36]. Consistent reporting of HRQoL outcomes, together with the conduct of prospective randomised studies evaluating the addition of PROs to conventional radiological and safety endpoints, is warranted to determine their impact on patient outcomes and trial efficiency, and to inform EPCTs designs, including phase transitions, clinical decisions, and initiation of supportive measures for patients. Adoption of HRQoL monitoring may enhance patient trust and transparency in EPCT participation, through demonstration that subjective experiences matter alongside objective tumour metrics, potentially improving recruitment and retention in early-phase studies—a persistent challenge worldwide. A prospective randomised study (PEARLER2) is currently underway to assess HRQoL, in the form of electronic PROs collected at trial review visits with real-time clinician access, to evaluate its impact on clinical decision-making and patient support activities in trials.

## 5. Conclusions

This study demonstrated that RECIST response does not reliably correlate with the changes to HRQoL in cancer EPCTs, with patients showing declines in their global health scores despite having stable or responding disease. Integration of PROs or HRQoL as an endpoint alongside other traditional safety and RECIST criteria should be considered in order to fully reflect the impact of investigative therapy on cancer patients, with implications on trial designs and clinical decisions.

## Figures and Tables

**Table 1:** Characteristics of evaluable cohort.

Characteristics	Value *n* (%)
Total	74
Median age [IQR; range]	64 [53.5–69.8; 25–83]
Sex	
Male	43 (58)
Female	31 (42)
Therapy type	
Targeted therapy	45 (61)
Immuno-oncology	29 (39)
Best RECIST	
Complete Response (CR)	0
Partial Response (PR)	15 (20.3)
Stable Disease (SD)	39 (52.7)
Progressive Disease (PD)	20 (27)

## Data Availability

The datasets for this manuscript are not publicly available, but requests to access the datasets should be directed to Jessie Nguyen (jessie.nguyen@health.nsw.gov.au) or Udit Nindra (udit.nindra@health.nsw.gov.au).

## Supplementary Materials

The following supporting information can be downloaded at: https://www.mdpi.com/article/10.3390/cancers18081242/s1, File S1: EORTC QLQ-C30 questionnaires; Table S1: Summary of variables, coding, and distribution supporting statistical methodology for cross-sectional analysis of ΔGHS vs RECIST response; Table S2: Multilevel Model Estimates: Adjusted Means, 95% Confidence Intervals, and Between-Group Comparisons (Exploratory Longitudinal Analysis).

## Abbreviations

The following abbreviations are used in this manuscript:

EPCT	Early-phase clinical trial
RECIST	Response Evaluation Criteria in Solid Tumours
PRO	Patient-reported outcomes
HRQoL	Health-related quality of life
CTCAE	Common Terminology Criteria for Adverse Events
