# Supplementary material for: Measuring What Matters: RECIST Response Does Not Predict HRQoL in Early-Phase Clinical Trials
Source: Cancers (Basel). 2026 Apr 14;18(8):1242. doi: 10.3390/cancers18081242 (PMC13115314; doi:10.3390/cancers18081242)
Supplement: Supplementary file 1 [file cancers-18-01242-s001.zip › Supplementary tables.pdf]

Table S1. Summary of variables, coding, and distribution supporting statistical methodology for cross-sectional analysis of ΔGHS vs RECIST response.

**Variable**	**Type**	**Coding / Definition**	**Distributions**
ΔGHS (Global Health Status change)	Continuous (non-normal)	Follow-up GHS – baseline GHS (EORTC QLQ-C30)	Median 0; IQR –16.7 to 8.3
RECIST response	Ordinal categorical	CR = complete response; PR = partial response; SD = stable disease; PD = progressive disease	PR: 15 (20.3%); SD: 39 (52.7%); PD: 20 (27.0%)
Therapy type	Binary categorical	Targeted therapy vs immuno-oncology	Targeted: 45 (61%); Immuno-oncology: 29 (39%)
Age	Continuous	Age at enrolment (years)	Median 64; range 25–83
Sex	Binary categorical	Male / Female	Male: 43 (58%); Female: 31 (42%)

ΔGHS demonstrated a non-normal distribution with wide variability, supporting the use of non-parametric statistical methods.

Table S2. Multilevel Model Estimates: Adjusted Means, 95% Confidence Intervals, and Between-Group Comparisons (Exploratory Longitudinal Analysis).

Best RECIST category	SD (Mean, 95% CI)	PR (Mean, 95% CI)	PD (Mean, 95% CI)	SD vs PD p-value	PR vs PD p-value
Cycle 1	70.4 (65.2, 75.7)	77 (66.6, 87.3)	65 (57, 73)	0.2654	0.0724
Cycle 2	69.4 (64.6, 74.2)	75.3 (65.8, 84.8)	60.4 (53.1, 67.7)	0.0442	0.0153
Cycle 3	68.4 (63.7, 73)	73.6 (64.5, 82.7)	55.8 (47.3, 64.3)	0.0114	0.0054
Cycle 4	67.3 (62.5, 72.1)	72 (62.8, 81.2)	51.2 (40.2, 62.2)	0.0087	0.0048
Cycle 5	66.3 (61, 71.6)	70.3 (60.5, 80.1)	46.6 (32.5, 60.8)	0.0108	0.0072
Cycle 6	65.3 (59.3, 71.3)	68.6 (57.8, 79.5)	42 (24.4, 59.6)	0.0144	0.0118
